# Trovafloxacin attenuates neuroinflammation and improves outcome after traumatic brain injury in mice

**DOI:** 10.1186/s12974-018-1069-9

**Published:** 2018-02-13

**Authors:** Charu Garg, Joon Ho Seo, Jayalakshmi Ramachandran, Ji Meng Loh, Frances Calderon, Jorge E. Contreras

**Affiliations:** 1 Department of Pharmacology, Physiology and Neurosciences, New Jersey Medical School, Rutgers University, 185 South Orange Ave, Newark, NJ 07103 USA; 2 Department of Mathematical Sciences, New Jersey Institute of Technology, University Heights, Newark, NJ 07102 USA

**Keywords:** Brain injury, Neuroinflammation, Microglia, Pannexin, Hemichannel

## Abstract

**Background:**

Trovafloxacin is a broad-spectrum antibiotic, recently identified as an inhibitor of pannexin-1 (Panx1) channels. Panx1 channels are important conduits for the adenosine triphosphate (ATP) release from live and dying cells that enhances the inflammatory response of immune cells. Elevated extracellular levels ATP released upon injury activate purinergic pathways in inflammatory cells that promote migration, proliferation, phagocytosis, and apoptotic signals. Here, we tested whether trovafloxacin administration attenuates the neuroinflammatory response and improves outcomes after brain trauma.

**Methods:**

The murine controlled cortical impact (CCI) model was used to determine whether in vivo delivery of trovafloxacin has anti-inflammatory and neuroprotective actions after brain trauma. Locomotor deficit was assessed using the rotarod test. Levels of tissue damage markers and inflammation were measured using western blot, qPCR, and immunofluorescence. In vitro assays were used to evaluate whether trovafloxacin blocks ATP release and cell migration in a chemotactic-stimulated microglia cell line.

**Results:**

Trovafloxacin treatment of CCI-injured mice significantly reduced tissue damage markers and improved locomotor deficits. In addition, trovafloxacin treatment significantly reduced mRNA levels of several pro-inflammatory cytokines (IL-1β, IL-6, and TNF-α), which correlates with an overall reduction in the accumulation of inflammatory cell types (neutrophils, microglia/macrophages, and astroglia) at the injury zone. To determine whether trovafloxacin exerted these effects by direct action on immune cells, we evaluated its effect on ATP release and cell migration using a chemotactic-stimulated microglial cell line. We found that trovafloxacin significantly inhibited both ATP release and migration of these cells.

**Conclusion:**

Our results show that trovafloxacin administration has pronounced anti-inflammatory and neuroprotective effects following brain injury. These findings lay the foundation for future studies to directly test a role for Panx1 channels in pathological inflammation following brain trauma.

**Electronic supplementary material:**

The online version of this article (10.1186/s12974-018-1069-9) contains supplementary material, which is available to authorized users.

## Background

Trovafloxacin is a fluoroquinolone antibiotic that exerts bactericidal activity by inhibiting prokaryotic topoisomerase enzymes, which are important for cellular division [1]. Recently, trovafloxacin was demonstrated to target human pannexin 1 (Panx1) channels at therapeutic concentrations reached in blood plasma [2]. Studies in mice have shown that Panx1 inhibition by trovafloxacin leads to dysregulated fragmentation of dying cells and blockade of ATP release [2]. Panx1 channels are large transmembrane pores that, besides ions, are permeable to small molecules such as ATP; they are expressed in various cell types [3, 4]. Recently, Panx1 channels have emerged as important players in response to injury and inflammation [5–7]. ATP release via Panx1 channels enhances inflammatory responses in peripheral immune cells and is implicated in the activation of the inflammasome [4, 6, 8]. Additionally, Panx1 channels expressed in endothelial cells can regulate the acute vascular inflammation by potentiating leukocyte emigration via ATP release [9]. In the brain, neuronal Panx1 channel activation during ischemia or cortical spreading depression is thought to be an important mechanism for mediating neuronal dysfunction and death [10–13]. Although it is likely that Panx1 channels also contribute to neuroinflammatory responses upon brain injury, their potential as therapeutic targets in traumatic brain injury (TBI) remains elusive.

The primary damage induced by mechanical brain trauma results in necrotic death of neurons, glial cells, and blood vessels [14–16]. The dying tissue produces damage-associated molecular pattern molecules (DAMPs, including ATP), which initiate and maintain an inflammatory response. The neuroinflammatory response is characterized by activation and migration of microglia and glial cells, leukocyte infiltration, and upregulation of inflammatory mediators [17, 18]. Elevated extracellular levels of ATP released upon injury have been shown to enhance the inflammatory response [19, 20]. ATP activates the purinergic pathway in inflammatory cells, thus playing an important role in migration, proliferation, phagocytosis, and apoptotic signals [21–24]. There is compelling evidence demonstrating that Panx1 channels, in part, represent a cellular mechanism for ATP release to the external milieu during inflammation [7, 25].

By taking advantage of previous work assessing the pharmacological properties of trovafloxacin in mice [2, 26, 27], we aimed to evaluate the potential role of trovafloxacin administration on curtailing inflammation in the controlled cortical impact (CCI) model of TBI. We found that in vivo administration of trovafloxacin significantly attenuated the inflammatory response in CCI injured mice. In addition, it decreased tissue damage and improved locomotor deficits. Our results also indicate that trovafloxacin diminish the accumulation of microglia and macrophages at the injury zone. In vitro studies showed that trovafloxacin as well as other Panx1 channel blockers inhibited ATP release and cell migration of a stimulated microglia cell line. We propose that a reduced number of pro-inflammatory cells at the injury site in trovafloxacin treated mice might be related to lesser migration and could contribute to improve outcomes after TBI.

## Methods

### Animal handling and CCI

All procedures were performed in accordance with the institutional guidelines and approved by the Institutional Animal Care and Use Committee of Rutgers-New Jersey Medical School. C57BL/6 mice (Charles River, USA) were housed two per cage during pre- and post-operative procedures with a 12-h light-dark cycle with ad libitum access to water and chow.

Ten-week-old-male mice were subjected to CCI injury using the stereotaxic impactor Impact One™ (Leica Biosystems, USA). Animals were secured in a stereotaxic frame and anesthetized with isoflurane (induction at 3% and maintenance at 2%) administered through a nose mask. A midline incision was made over the skull. A unilateral craniectomy was performed between Bregma and Lambda using a hand drill with a 5-mm-diameter trephine. Special care was taken to prevent any damage to the dura mater, therefore assuring it was intact after each craniotomy. Animals were impacted using a 4.0-mm stainless steel flat impactor tip, at stereotaxic coordinates AP − 2.26, ML + 2.0 and 0.65 mm deep at a rate of 4.0 m/s and a dwell time of 200 ms, at an angle of 0.4°. After injury, any bleeding was cleaned up, the incision was sutured with clips, and the animals were immediately removed from anesthesia. Post-surgery, the mouse was placed on its back in a cage, which was set over a heating pad. The recovery of each mouse was observed until they were standing on their four paws. Sham animals went through the same procedures as CCI-injured animals, including anesthesia and skin incision over the skull, but not craniotomy, as it has been shown that the craniotomy procedure alone stimulates production of pro-inflammatory cytokines at 24 h after surgery [28], which would confound our analyses. The stock of trovafloxacin (100 mM) was prepared in DMSO and was then diluted to 1:10 in saline. Trovafloxacin-treated group was given intraperitoneal injections of 60 mg/kg at 1, 24, and 48 h post-CCI injury. Non-treated CCI-injured animals received vehicle only.

### Total RNA extraction, reverse transcription, and real-time PCR (RT-qPCR)

Injured cortex was carefully dissected from the ipsilateral hemisphere using an adult mouse brain slicer. Total RNA was isolated using Trizol (Thermo Fisher scientific, USA) according to the manufacturer’s protocol. Two micrograms of RNA was reverse transcribed using High Capacity RNA-to-cDNA kit (Thermo Fisher scientific, USA). TaqMan® Universal PCR Master Mix and TaqMan® FAM™ conjugated primers (Thermo Fisher Scientific, USA) were used to evaluate mRNA using the ABI 7500 Sequence Detection System (Applied Biosystems, USA). mRNA expression was normalized to GAPDH as endogenous control, and the relative fold difference in expression was calculated using the comparative 2^−ΔΔCT^, a widely used method to present relative expression respect to controls (shams) [29, 30]. The following primer genes were assessed: IL-1β (Accession#Mm00434228_m1), TNF-α (Accession#Mm00443258_m1), IL-6 (Accession #Mm00446190_m1), MPO (Accession #Mm01298424_m1), GAFP (Accession #Mm01253033_m1), CD68 (Accession #Mm03047343_m1), and Iba1 (Accession #Mm00520165_m1). GAPDH (Accession #Mm99999915_g1) was used as an endogenous control. The ΔΔCT method was used to calculate the relative gene expression levels respect to shams.

### Total protein extraction and western blot analysis

Brain tissues enclosing the injury were carefully dissected from the ipsilateral cortex under a dissecting microscope and then homogenized in buffer containing M-PER Mammalian protein extraction reagent 5 mM Na_3_VO_4_, 1 mM NaF, 1 mM Na_2_P_2_O_7_, 1 mM Bezamidine, 5 mM EDTA, and Halt Protease Inhibitor Cocktail (Thermo Fisher scientific, USA). Protein concentrations were estimated using a BCA kit (Pierce, USA). Equal amounts of protein (20 μg) per sample was separated on 4–20% gradient gels (Bio-Rad, USA) and run under the same experimental conditions, transferred to PVDF membranes, and probed with the following antibodies: GFAP (Cell Signaling, USA), CD68 (Abcam, USA), α–ΙΙ spectrin (Santa Cruz Biotechnology, USA), MMP-9 (NeuroMab, USA), and GAPDH (Cell Signaling, USA). Blots were developed using enhanced chemiluminescence, and densitometric analysis was performed using Fuji Images or Bio-Rad Image Lab software.

### Cell culture

BV-2 cell line was previously generated by others through  infection of murine primary microglial cells with a v-raf/v-myc oncogene carrying retrovirus [31]. This cell line has been found to retain some of the morphological, phenotypical, and functional properties of freshly isolated microglial cells and is considered immortalized microglial cells [32]. BV-2 cells were seeded at a density of 7.5 × 10^5^ cells/ml and maintained in DMEM/F-12 supplemented with 5% FBS, penicillin 100 IU/ml, and streptomycin 100 μg/mL. Cell cultures were kept in a cell incubator at 37 °C with 95% air and 5% CO_2_ and saturated humidity.

### ATP release measurements

Extracellular ATP release was measured using the ATP bioluminescence assay kit (Molecular Probe, USA) following the manufacturer’s instructions. BV-2 cells were seeded in 24-well culture plates at a cell density of 1.5 × 10^5^ cells/well in serum-free DMEM media supplemented with 4 mM L-glutamine. After 24 h, cells were treated with C5a (10 nM) in the presence or absence of Panx1 channel blockers trovafloxacin (1 μM), Brilliant Blue FCF (5μM) or ^10^Panx1 (200 μM) in serum-free DMEM medium. Extracellular ATP was measured before C5a stimulation and every 10 min thereafter. At the indicated time points, 10 μl aliquots from a total volume of 300 μl were collected from the culture supernatants for ATP determinations.

### Transmigration assay

Migration assay was performed using 24-well plates and 8.0-μm pore size transwell inserts (Corning Costar, NY, USA). BV-2 cells suspended in serum-free medium were seeded at a density of 1.5 × 10^5^ cells/ml/well in the upper chamber of the transwell insert. Cells were allowed to attach overnight. Then, the cells were stimulated with C5a (10 nM) or ATP (200 μM) in the presence or absence of pannexin channel blockers trovafloxacin (1 μM), Brilliant Blue FCF (5μM) or ^10^Panx1 (200 μM). At 4 or 24 h post treatment, cells were fixed with 4% paraformaldehyde (PFA) and stained with 0.05% crystal violet. BV-2 cells that did not migrate were removed from upper chamber by wiping with cotton swabs. Cells that migrated to the bottom of the filters were quantified from at least 5 images taken from different fields taken at 20× using an Olympus AX70 microscope.

### Brain sample preparation for histology and immunofluorescence

For morphological analysis, CCI-injured mice were anesthetized with ketamine/xylazine at 6 days post-injury and transcardially perfused with RPMI media containing heparin (1000 USP units/ml) at a rate of 4 ml/min followed by 4% PFA in PBS, pH 7.4 at a rate of 5 ml/min. Once the animals were fixed, mice were decapitated and the whole brains were removed, taking care to keep the contusion region intact. After fixation, brains were immersed in 30% sucrose for 24 h and frozen − 80 °C until sectioning. Twenty micrometer coronal sections were made from whole brains using a cryostat.

#### Immunostaining

Brain sections were taken at room temperature for 20 min. Then, they were washed twice with tris buffer 1× (TBS), pH 7.4 and permeabilized with 0.3% triton X-100 in TBS in a humid chamber at room temperature for 30 min. Sections were washed again with TBS and incubated in TBS buffer containing 10% BSA, 10% Normal Donkey Serum, pH 7.4 (TDB) in humid chamber at room temperature for 1 h. Primary antibody against CD68 (Bio-Rad, USA) diluted 1:300 in TDB diluent containing 20% TDB solution, 0.2% triton X-100, and 80% TBS pH 7.4 were applied to the slides and kept in a humid chamber at 4 °C for 12 h. The sections were washed for 5 min in TBS and then incubated with secondary antibodies (Cy3™ or Alexa Fluor 488 Donkey Anti-Rabbit IgG) from Jackson Immunoresearch (West Grove, USA) diluted 1:300 in TDB diluent were applied on the sections at room temperature for 2 h. Tissue slides were washed with 1× TBS buffer for 5 min. Samples were counterstained with 4′,6-diamidino-2-phenylindole (DAPI) for nuclear staining at 1:10,000 dilution for 10 min at room temperature in a humid chamber. Slides were rinsed twice with 1× TBS for 5 min each. Glass cover slips were mounted on the samples on glass slides with fluorescent mounting medium containing anti-fade (Gelvatol containing DABCO). Slides were left to dry at room temperature for 24 h and then stored at 4 °C. Images were captured using 10× objective in up-right fluorescence BX51 Olympus microscope and injury areas were evaluated using ImageJ software.

### Quantification of areas for high-density immunofluorescence of CD68 positive microglial/macrophage cells

The core of the injury was morphologically identified as the region with the deepest damage in serial sections. At 6 days post injury, we identified a lesion zone with a bottom of approximately 600-μm diameter centered around the stereotactic coordinates of the epicenter (AP − 2.26, ML 2.0). This lesion zone was filled with a high density of CD68+ cells. The areas of high-density CD68+ cells were measured using the ImageJ 1× software [33]. Using a slide scale under 4× objective in the fluorescence microscope, the pixels equivalent to 1 mm (388 pixels) were identified and set to scale in the ImageJ software. Using the polygon selection tool, high-density immunofluorescence of CD68+ cells were selected to enclose an area of injury. This area was measured in square millimeter and plotted against the distance from Bregma. To keep the fluorescence intensity levels uniform across all slides, they were stained all at the same time and images were acquired using the same settings and time exposure to minimize threshold bias.

### Rotarod performance test

Mice were placed on a rotarod machine (IITC Life Sciences, USA) that has an accelerating rotating cylinder suspended over a platform. When the animal falls the platform is displaced and the machine records the latency in seconds for the animal to fall. This acquisition phase was performed at days 1, 3, and 5 after injury, (3 trials per day). Mice were trained in the rotarod for 3 days before CCI injury; last training day was considered the baseline testing. Sham, CCI vehicle, and CCI + trovafloxacin mice were evenly grouped on the basis of their average latency to fall.

### Statistical analysis

One-way or two-way ANOVA followed by Tukey’s HSD (honest significant difference) test was used to determine statistical significance in migration assay and western blot analysis, respectively. Linear regression analysis was performed for time series of measurements using treatment and the interaction between treatment and time as factors. These analyses include ATP assays, RT-qPCR, and behavior. For immunofluorescence of CD68 analysis, linear regression was performed using treatment and the interaction of treatment and distance from Bregma as factors. Analyses were performed using SPSS 15.0 (IBM) or *R* statistical software. All experiments were performed at least three times. The values were expressed as the means ± SEM. The differences with *p* < 0.05 were considered statistically significant. Values are represented as means ± SEM. Differences with *p* values < 0.05 were considered statistically significant.

## Results

### Trovafloxacin improves locomotor recovery and reduces tissue damage in CCI-injured mice

**Fig. 1:**
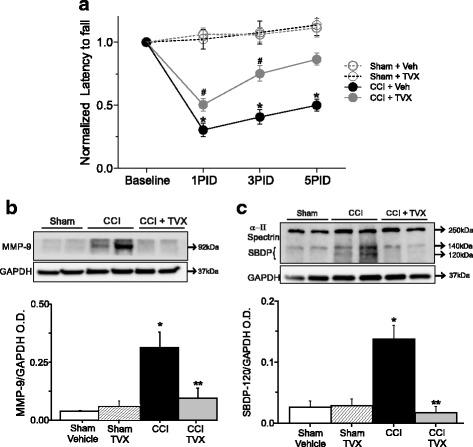
Trovafloxacin treatment improves locomotor behavior and attenuates brain damage. **a** The rotarod test was used to evaluate latency (time) to fall in sham treated with vehicle (gray open circles), sham treated with trovafloxacin (TVX, black open circles), CCI-injured mice treated with vehicle (gray closed circles), or trovafloxacin (TVX, black closed circles) for up to 5 days post-CCI. Latency to fall was normalized to the baseline behavior for each group. Values are represented as mean ± SEM (*n* = 8 per group). Linear regression analysis was used to determine statistical difference between different groups at each time point (*p* values calculated for treatment and treatment*time). *p < 0.05, sham vehicle vs. CCI + vehicle; #*p* < 0.05 sham vehicle vs. CCI + TVX. Identical *p* values at their respective PDI were found for sham + TVX vs. CCI vehicle or CCI + TVX (not symbols display). **b** Representative western blots images showing MMP-9 protein levels at 6 days post-injury in sham treated with vehicle and CCI-injured mice treated with vehicle or trovafloxacin. Bottom western blot corresponds to the GAPDH, which was used as housekeeping gene. Note that sham mice treated with TVX are not display in the western blots. Graph shows to densitometric quantification of MMP-9 normalized to GAPDH values. Values are represented as mean ± SEM (*n* = 5 per group). **c** α–ΙΙ spectrin and SPDBs (140 and 120 kDa) expression levels at 6 days post-injury in sham and CCI-injured mice treated with vehicle or trovafloxacin. Bottom western blot shows GAPDH. Note that sham mice treated with TVX are not displayed in the western blots. Graph shows densitometric quantification of SPDB 120 kDa normalized to GAPDH expression. Values are represented as mean ± SEM. (*n* = 5 per group). Two-way ANOVA followed by Tukey’s HSD test was used to determine statistical significance. *p < 0.01, sham vehicle vs. CCI group; **p < 0.01, CCI vehicle vs. CCI + TVX. No differences were found between sham vehicle and sham TVX (see also Additional file 1: Figure S1)

We conducted locomotor behavioral analysis using the rotarod coordination test at 1, 3, and 5 days post-injury in sham and CCI-injured mice treated with vehicle or trovafloxacin. Vehicle-treated CCI-injured mice showed a significant decrease in the latency to fall at 1 day post-injury (Fig. 1a), and their performance remained greatly impaired at 3 and 5 days post injury. Conversely, trovafloxacin-treated mice showed longer latencies to fall as compared to the vehicle-treated CCI-injured group. At 3 and 5 days post injury, trovafloxacin-treated animals display a recovery in locomotor performance (Fig. 1a). Importantly, sham animals treated with vehicle or trovafloxacin do not display differences in locomotor activity.

To determinate whether the improvements observed in the behavioral outcome of trovafloxacin-treated mice correlate with neuroprotective actions after CCI, we analyzed protein levels of matrix metallopeptidase 9 (MMP9) and neuronal α*–*ΙΙ spectrin breakdown products (SBDPs) in CCI-injured mice treated with vehicle or trovafloxacin. MMP9 and SBDPs are general biomarkers for brain injury, such as TBI, in rodents and humans [34, 35]. Figure 1b shows that MMP-9 levels were sixfold higher in the CCI-injured group as compared to the sham group. Conversely, mice treated with a daily *i.p.* injection of trovafloxacin for up to 3 days post CCI showed a significant reduction in MMP-9 levels when compared to vehicle-treated CCI animals. The protein levels of full-length α–ΙΙ spectrin (250 kDa) and SBDP (140 and 120 kDa) were also examined at the cortex at 6 days post CCI (Fig. 1c). Full α–ΙΙ spectrin and SBDP 140 kDa remain unchanged in all three groups; conversely, the levels SBDP 120 kDa levels were noticeable higher in vehicle-treated CCI mice when compared to sham and trovafloxacin-treated mice. This suggests that brain injury-induced neuronal proteolysis in CCI-injured mice is reduced by trovafloxacin treatment. We also did not find differences in biomarker levels in sham mice treated with vehicle or trovafloxacin (Additional file 1: Figure S1).

**Fig. 2:**
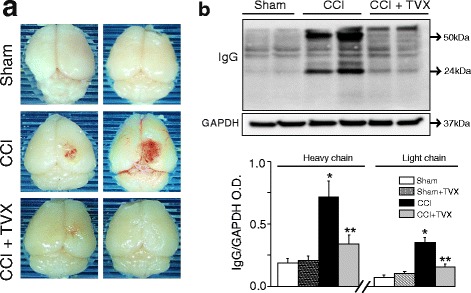
Trovafloxacin treatment attenuates blood brain barrier leakage in CCI-injured mice. **a** Representative images of perfused and fixed mice brains from sham and CCI-injured mice treated with vehicle or trovafloxacin (TVX) after 6 days post-CCI. **b** Representative western blot showing IgG protein levels from injury of sham and CCI-injured mice treated with vehicle or TVX 6 days post-injury. Bottom western blot corresponds to GAPDH. **b** Densitometric quantification of IgG levels for each group (*n* ≥ 5 per group) relative to their corresponding GAPDH expression levels. Note that sham mice treated with TVX are not displayed in the western blots. Values are represented as mean ± SEM. Two-way ANOVA followed by Tukey’s HSD test was used to determine statistical significance. *p < 0.01, sham vs. CCI group; **p < 0.01, CCI vehicle vs. CCI + TVX. No differences were found between sham vehicle and sham TVX (see also Additional file 1: Figure S1)

In addition to biochemical analysis of TBI biomarker protein levels, visual observation of fixed CCI-injured brains from mice treated with vehicle or trovafloxacin clearly indicated that trovafloxacin treatment reduces damage. Qualitative analysis shows that the size of a CCI-induced hematoma is smaller in trovafloxacin-treated mice as compared to those treated with vehicle (Fig. 2a). To confirm that trovafloxacin treatment protects the integrity of the blood brain barrier after trauma, we performed western blot analysis against immunoglobulin G (IgG) chains to assess its infiltration into the parenchyma in ipsilateral brains from sham, vehicle, and trovafloxacin-treated mice 6 days post injury. Figure 2b shows that while the levels of heavy and light chain of IgG are not noticeable in sham cortex, both proteins are significantly elevated in vehicle treated CCI-injured mice. Conversely, those CCI-injured mice treated with trovafloxacin show a twofold decrease in the protein levels of heavy and light IgG chains as compared to vehicle-treated CCI mice (Fig. 2b). Taken together, our results indicate that treatment with trovafloxacin improves tissue integrative and behavioral outcomes after CCI injury.

### Trovafloxacin decreases mRNA levels of pro-inflammatory cytokines and activated innate immune and glia cells after CCI

**Fig. 3:**
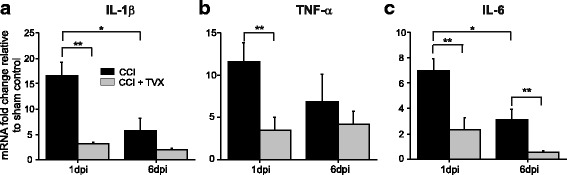
Pro-inflammatory cytokines mRNA levels are decreased in trovafloxacin-treated CCI mice. RNA was isolated from ipsilateral cortex from sham and CCI-injured mice treated with vehicle or trovafloxacin (TVX). Gene expression levels for **a** IL-1β, **b** TNF-α, and **c** IL-6 were measured at 1 and 6 days post-injury by qPCR. Values are expressed as mean fold change (± SEM) relative to sham (*n* = 5), and GAPDH was used as endogenous control. Linear regression analyses were used to determine statistical significance. (*p* values calculated for dpi and treatment, for IL-1β, TNF-α, and IL-6 separately). *p < 0.01, CCI 1 dpi vs. CCI 6 dpi, **p < 0.05, CCI vehicle vs. CCI + TVX

Neuroinflammation is a major component of secondary traumatic brain injury, which contributes to the ongoing neurodegeneration associated with brain damage. The levels of pro-inflammatory cytokines are significantly upregulated in response to TBI and play central roles in the initiation and propagation of the inflammatory response [36]. We performed RT-qPCR to evaluate whether treatment with trovafloxacin reduces mRNA levels of the pro-inflammatory cytokines IL-1β, TNF-α, and IL-6 after CCI injury. Figure 3 shows that mRNA levels of IL-1β, TNF-α, and IL-6 are increased several fold in the vehicle-treated CCI group at 1 day post-CCI when values were normalized with respect to sham values. Subsequently, we observed a reduction in mRNA levels of all three cytokines at 6 days post-CCI when compared to 1 day post-CCI. Conversely, trovafloxacin treatment (i.p. injection, 60 mg/kg) markedly reduced IL-1β, TNF-α, and IL-6 mRNA by at least 2.5 fold at 1 day post-injury when compared to vehicle-treated CCI animals (Fig. 3a–c). IL-6 mRNA levels remained significantly reduced in trovafloxacin treated mice 6 days post-injury as compared to vehicle-treated CCI mice. Similar results were found for all three cytokines tested 1 day post-injury in mice treated with another Panx1 channel blocker, the food dye Brilliant Blue FCF [37] (Additional file 2: Figure S2).

**Fig. 4:**
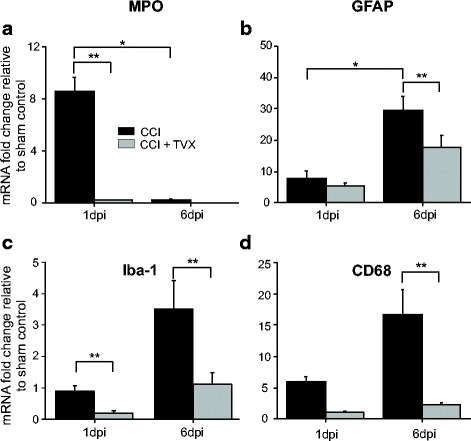
Trovafloxacin treatment attenuates the expression levels of markers for leukocytes and glia cells in the injured brain. RNA was isolated from ipsilateral cortex from each group, and gene expression levels were determined by qPCR at 1 and 6 days post-injury: **a** myeloperoxidase (MPO), marker of neutrophils; **b** glial fibrillary protein (GFAP), marker of astrocytes; **c** ionized calcium-binding adapter molecule 1 (Iba-1), marker of microglia; and **d** cluster of differentiation 68 (CD68), marker of activated microglia. Values are expressed as mean fold change (± SEM) relative to sham, and GAPDH was used as endogenous control. Linear regression analyses were used to determine statistical significance. (*p* values calculated for dpi and treatment, for MPO, GFAP, Iba-1, and CD68 separately). *p < 0.01, CCI 1 dpi vs. CCI 6 dpi, **p < 0.05, CCI vehicle vs. CCI + TVX

Leukocyte infiltration, microglia accumulation, and glial cell proliferation at the injury zone are key components of the neuroinflammatory response after brain trauma. Accordingly, we examined the extent of neutrophil infiltration (myeloperoxidase; MPO), levels of activated astrocytes (glial fibrillary acidic protein, GFAP), and accumulation of microglia/macrophage cells (ionized calcium-binding adapter molecule 1, Iba1; and cluster of differentiation 68, CD68) in the injury site. mRNA levels of these markers are known to increase after TBI [14, 38–41]. Figure 4a shows that mRNA levels of MPO in CCI-injured animals significantly increased with respect to sham animals; however, a single trovafloxacin i.p. injection at 1 h post-CCI significantly reduced MPO expression levels. mRNA levels of MPO were decreased in both vehicle and trovafloxacin-treated CCI mice 6 days post-injury. This is consistent with previous observations showing that the presence of neutrophils at the injury zone diminished 6 days post-CCI [42]. In contrast, microglia and macrophage accumulation, as well as astrogliosis, have been shown to steadily increase up to 6 days post-injury [43]. In line with this, Fig. 4b–d shows that expression levels of GFAP, Iba1, and CD68 in CCI-injured animals increased in a time-dependent manner when compared to sham animals. Strikingly, trovafloxacin administration significantly attenuated the levels of these markers in CCI-injured animals by at least twofold (Fig. 4b–d). Together, our results indicate that trovafloxacin treatment reduced the neuroinflammatory response triggered by CCI.

### Microglia and macrophage accumulation is reduced in CCI -injured mice treated with trovafloxacin

**Fig. 5:**
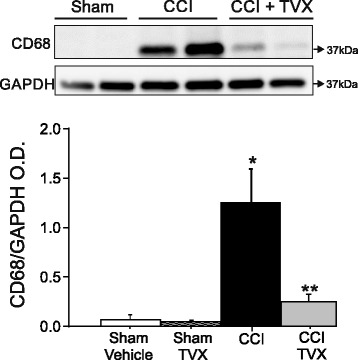
Trovafloxacin treatment reduces CD68 protein expression levels at 6 days post-injury. Representative western blots showing CD68 protein levels expressions at 6 days post-injury in sham or CCI-injured mice treated with vehicle or trovafloxacin (TVX). Graph shows densitometric quantification of CD68 is represented in each group (*n* = 5 per group). Values are represented as mean ± SEM. Two-way ANOVA followed by Tukey’s HSD test was used to determine statistical significance. *p < 0.01, sham vs. CCI group; **p < 0.01, CCI vehicle vs. CCI + TVX

Sustained activation of pro-inflammatory microglia is one of the detrimental processes leading to neuronal damage during brain injury [44]. CD68 is a transmembrane glycoprotein that expresses in activated microglia and macrophages serving as a marker of inflammation. To further demonstrate that trovafloxacin administration diminishes accumulation of microglia and macrophage cells at the injury site, we analyzed protein levels and expression of CD68 in the ipsilateral hemisphere using western blot and immunofluorescence, respectively. Previous work has shown that CD68 expression levels reach maximum at 6 days post-injury [41]; consistent with this, Fig. 5 shows a significant increase in CD68 protein levels in CCI-injured animals when compared to the sham group at this time point. Trovafloxacin-treated CCI mice, however, display a fourfold decrease in CD68 protein levels at the ipsilateral cortex compared to vehicle-treated animals (Fig. 5). This result is in agreement with RT-qPCR data, supporting the notion that trovafloxacin reduces accumulation of microglia and macrophage cells.

**Fig. 6:**
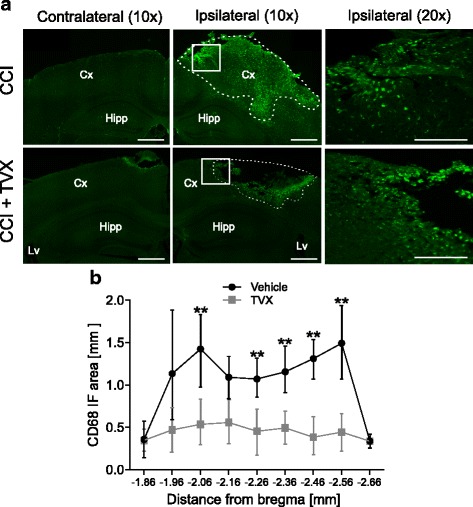
Microglia/macrophage accumulation is reduced in trovafloxacin-treated CCI mice. Mice that received CCI injury where treated with trovafloxacin (TVX) or vehicle and were sacrificed at 6 dpi for histology analysis. **a** Representative images of CD68 immunoreactivity of brain sections from non-treated or TVX-treated CCI-injured mice at the core of the injury (AP − 1.96 to AP − 2.56). These areas were examined in the same anatomical location for each animal. Scale bar = 0.5 mm. Dotted lines demarcated high-density areas of CD68+ staining. **b** Correspond to the quantification of high-density areas of CD68+ cells (*n* = 5 per group). Error bars indicate SE of the mean. Linear regression analysis was performed to analyze statistical differences. (*p* values calculated for distance from Bregma and treatment). **p < 0.05 CCI vehicle vs. CCI + TVX

To further confirm whether accumulation of microglia/macrophages from CCI-injured animals is attenuated by trovafloxacin, we quantified the high-density areas of CD68 immunoreactive cells in the ipsilateral cortex from vehicle-treated and trovafloxacin-treated CCI-injured mice at 6 days post-injury (Fig. 6). We analyzed the core of the injury, which was identified in serial sections stained with DAPI as a cavity with a flat bottom of approximately 600-μm diameter centered on the epicenter at AP − 2.26 and ML 2.0 in CCI-injured mice. The lesion or cavity was filled with CD68+ cells and was visibly less pronounced in trovafloxacin-treated animals (Fig. 6a, compared ipsilateral hemispheres). Quantification of the high-density CD68+ cells area show that administration of trovafloxacin in CCI-injured mice significantly attenuated the accumulation of microglia/macrophage cells at the core of the injury in the ipsilateral cortex (Fig. 6b) reducing the number of CD68+ cells by up to 50 % (AP − 2.06 and AP − 2.46).

### Trovafloxacin inhibits ATP release and microglial migration in vitro by inhibition of Panx1 channels

**Fig. 7:**
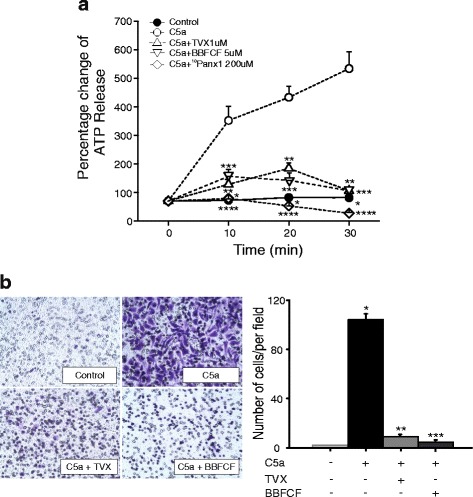
Trovafloxacin inhibits ATP release and migration in C5a-stimulated BV-2 cells. **a** BV-2 cells were stimulated with 10 nM of C5a in the presence or absence of Panx1 channel blockers, trovafloxacin (TVX, 1 μM), Brilliant Blue FCF (BBFCF, 5 μM), or ^10^Pnx1 (200 μM) for 30 min. Aliquots were collected from the medium every 10 min for ATP measurement. ATP was measured using the luciferin–luciferase assay. Values are represented as mean ± SEM. Percentage values were estimated with respect to basal ATP release before simulation at each condition. Each experiment was repeated at least three times in triplicates. Statistical difference was determined using linear regression with log(ATP) as the response. (*p* values calculated for treatment and treatment*time). *p < 0.05, C5a vs. control; **p < 0.05, C5a vs. TVX; ***p < 0.05, C5a vs. BBFCF, ****p < 0.05, C5a vs. ^10^Panx1. **b** Left panel shows representative images of BV-2 cells that were seeded in the upper compartment of the transwells for transmigration assay and treated with C5a in the absence or presence of TVX (1 μM) or BBFCF (5 μM). After 4 h, cells that had migrated to the bottom of the transwell were fixed, stained with crystal violet, and counted. The images are representative of the control treated with vehicle solutions, C5a, C5a + TVX, and C5a + BBFCF-treated cells. Graph corresponds to the quantification of transmigrated BV-2 cells after 4 h stimulation from images taken in five different fields at 20× per condition. Each experiment was repeated at least three times. One-way ANOVA with Tukey’s HSD test was used to measure statistical differences among groups. Values are expressed as mean ± SEM, *p < 0.05, control vs. C5a; **p < 0.05 C5a vs. C5a + TVX, or ***p < 0.05, C5a vs. C5a + BBFCF

Accumulation of activated microglia and macrophages in the ipsilateral cortex depends, at least in part, of microglia migration to the injury site and infiltration of monocytes. These processes are related to purinergic signaling and have also been linked to Panx1 channel activation, which acts as a conduit for ATP release in various cell types [9, 45]. Therefore, we next tested whether trovafloxacin attenuates acute ATP release and migration in a stimulated murine microglial cell line (BV-2 cells). Cells were stimulated with the complement component 5a (C5a), an extracellular soluble protein that forms part of a complement component activated upon tissue injury. C5a is a potent mediator of the innate immune response [46], and blockade of the C5a receptors have been shown to improve outcomes in rodent models of TBI [47]. Figure 7a shows that incubation of BV-2 cells (open circles) with C5a (10 nM) promotes extracellular ATP release as compared to non-stimulated cells (closed circles). Extracellular ATP release was significantly inhibited when C5a-stimulated BV-2 cells were co-incubated with 1 μM trovafloxacin (upright triangles), 5 μM BBFCF (upside down triangles), or 200 μM ^10^Panx1, a specific Panx1 mimetic blocker peptide (open diamonds). To reliably measure extracellular ATP levels in each experiment, these measurements were performed in the presence of 10 μM POM 1, a generic ecto-ATPase inhibitor that prevents breakdown of ATP. Importantly, the action of these different known Panx1 channel blockers on ATP release in this culture system strongly suggests that the ATP is release via Panx1 channels.

**Fig. 8:**
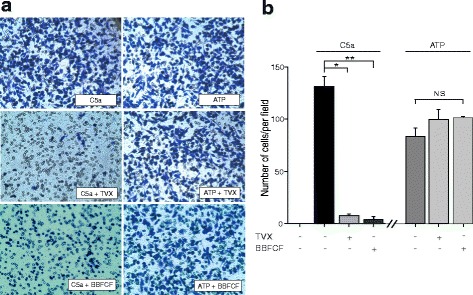
Trovafloxacin does not affect downstream of ATP signaling. **a** Representative images of BV-2 cells that were seeded for transmigration assay and stimulated with C5a (10 nM) or ATP (200 μM) for 24 h in the presence or absence of trovafloxacin (TVX, 1 μM) or Brilliant Blue FCF (BBFCF, 5 μM). Cells were fixed and stained with crystal violet. **b** Quantification of transmigrated BV-2 cells at 24 h from five different fields at 20× per condition. Each experiment was repeated at least three times. One-way ANOVA with Tukey’s HSD test was used to measure statistical differences among groups. Values are expressed as mean ± SEM. *p < 0.05, C5a vs. C5a + trovafloxacin. **p < 0.05, C5a vs. C5a + BBFCF. No significant changes in the number of migrated cells were observed between ATP, ATP + TVX, and ATP + BBFCF (*p* > 0.05)

Because extracellular ATP release promotes migration of microglial cells both in vitro and in vivo [9, 48], we assessed whether trovafloxacin treatment reduces C5a-induced BV-2 cell migration in a transwell migration assay. POM 1 was not used in these experiments because ATP byproducts also activate different purinergic receptors involved in chemotaxis [49]. Figure 7b shows lack of BV-2 cell migration in the absence of stimulation with C5a. Conversely, after a 4-h incubation with 10 nM C5a, a significant number of cells migrate through the pores, as detected by crystal violet staining (Fig. 7b). Co-incubation with 1-μM trovafloxacin or 5-μM Brilliant Blue FCF drastically reduced migration of BV-2 cells when compared with cells exposed to C5a alone (Fig. 7b). Quantitative analysis indicated that C5a treatment shows 131 ± 9 migrating cells per field versus 8 ± 2 or 3 ± 2 cells per field when BV-2 cells were co-incubated with trovafloxacin or Brilliant Blue FCF, respectively (Fig. 7b). A potential mechanism that could mediate this reduction in C5a-induced migration by trovafloxacin is blockade of Panx1 channels and thereby reduced ATP release. To confirm whether the reduction in the number of migrating BV-2 cells is due to the blockade of ATP release and not through downstream effects (i.e., trovafloxacin block of purinergic receptor signaling), we evaluated BV-2 cell migration by directly applying 200 μM ATP to the cell culture medium in the absence or presence of 1 μM trovafloxacin. No significant changes in the number of ATP-stimulated migrating BV-2 cells were observed in the presence or absence of trovafloxacin or Brilliant Blue FCF (Fig. 8). These data support the notion these blockers reduce extracellular ATP release potentially via the blockade of Panx1 channels, without affecting ATP signaling downstream of purinergic receptor activation.

## Discussion

In the present study, we found that administration of trovafloxacin to CCI-injured mice produced anti-inflammatory and neuroprotective effects and, importantly, ameliorated CCI-induced locomotor impairment. The beneficial effects of trovafloxacin treatment in this animal model of TBI are supported by (1) decreased tissue damage that correlated with improved locomotor behavioral outcomes; (2) significantly reduced mRNA levels of pro-inflammatory cytokines (IL-1β, IL-6, and TNF-α) at their corresponding expression peaks; (3) reduced mRNA levels of infiltrating neutrophils (MPO), reactive astrocytes (GFAP), microglia and macrophage cells (CD68, Iba-1), which was corroborated by immunofluorescence, and western blot analyses; and (4) in vitro assays demonstrating a trovafloxacin-dependent reduction in migration of stimulated microglial cell lines via blockade of extracellular ATP release.

Although trovafloxacin was originally described as a broad-spectrum antibiotic, it has been recently shown that it is a blocker for Panx1 channels. Thus, it is possible to hypothesize that Panx1 channel activation after brain trauma enhances the neuroinflammatory responses via ATP release. Other potential routes of ATP release that are involved in stroke-induced neurodegeneration [11, 50] implicated Cx43 hemichannels or Panx2 channels; however, these channels are not blocked by trovafloxacin [2]. Moreover, data from our laboratory indicate that trovafloxacin does not block other large ATP permeable pores including Cx26, Cx46, and CALHM-1 channels (unpublished results). It is important to note, however, that trovafloxacin was withdrawn from the market relatively soon after its release as a generic antibiotic due to the risk of hepatotoxicity [51]. Trovafloxacin-induced hepatotoxicity appears to occur in conjunction with episodes of inflammatory stress associated with high levels of TNF-α in blood plasma. For example, a single trovafloxacin dose of 80 mg/kg or greater caused hepatotoxicity in LPS-treated mice, but 1000 mg/kg trovafloxacin in mice non-treated with LPS did not exert toxic effects [26]. In the present study, we used daily doses of 60 mg/kg via *i.p* that was not extended for more than 3 days post-injury. Interestingly, this dosage was enough to attenuate TBI-induced neuroinflammatory events that peak at 6 days post-injury suggesting that early action of trovafloxacin is critical in affecting the later progression of the neuroinflammatory response.

Fluoroquinoline antibiotics including alatrofloxacin and trovafloxacin have previously been shown to have immunosuppression effects in infected monocytes and macrophages [52–54]; however, no mechanisms of actions have been described. More recent work has shown that trovafloxacin might inhibit α-adrenoreceptors and suppress the activation of the peroxisome proliferator-activated receptor alpha (PPARα) in the liver [55, 56]. While the blockade of α-adrenoreceptors by trovafloxacin seems to be mediated by direct interactions, the mechanism by which trovafloxacin suppresses PPARα activation is still unclear. The role of these two receptors in brain trauma has also been well documented; crucially, it has been shown that activation, but not inhibition, of these receptors is neuroprotective after brain injury [57–60]. For example, there is compelling evidence that activation of PPARα promotes anti-inflammatory and neuroprotective effects in several models of brain trauma [61–64]. Moreover, blockade of α-adrenoreceptors increases behavioral deficits in traumatic brain injury [57]. Therefore, it is unlikely that inhibition of α-adrenoreceptors and PPARα by trovafloxacin contributes to neuroprotection in our model of brain injury since, then, we would expect opposite results. Moreover, the fact that another Panx1 channel blocker like Brilliant Blue FCF has similar effects to trovafloxacin, at least at 1 day post-CCI supports the idea that trovafloxacin may have neuroprotective actions by inhibiting Panx1 channels. However, this hypothesis needs to be tested directly in future studies.

Several studies indicate that blockade or global deletion of Panx1 after stroke is neuroprotective [11–13, 65]. Panx1 is ubiquitously expressed in the brain, identified in both neurons and astrocytes. Also, leukocytes, microglia, and endothelial cells express Panx1 protein. Thus, it is possible that the beneficial effects exerted by trovafloxacin involve multiple neuronal and non-neuronal cell types. For instance, neuronal Panx1 activation via src-kinase has been recently shown to be deleterious in ischemia-induced excitotoxicity in vitro and in vivo [66, 67]. Moreover, endothelial Panx1 is also essential for leukocyte emigration in the acute inflammatory response by acting as a conduit for ATP release [9], whereas neuronal and astrocytic activation of Panx1 induces inflammasome activation in vitro [6].

Among the multiple cell types that could be targeted by trovafloxacin in our model of TBI, we focused on the accumulation of pro-inflammatory microglia and macrophages at the core of the injury site. In addition to cell proliferation, the accumulation of inflammatory cells requires infiltration of leukocytes (neutrophils and monocytes) and microglial migration. These events are mediated by activation of purinergic signaling via extracellular ATP and its byproducts [22]. Several sources for ATP release upon injury have been described; an important contributor is the Panx1 pathway activated by dying cells. These cells function as a signal beacon to direct or point innate immune cells towards apoptotic cell death activity [24, 45]. Autocrine release of ATP from infiltrating innate immune cells is also associated with Panx1 channel activation and might contribute to cellular migration [68]. Consistent with this idea, we found that trovafloxacin significantly reduced extracellular ATP release from C5a-stimulated BV-2 cells. It also prevented cell migration without affecting purinergic receptor activation and downstream signaling. Thus, our in vitro data may partially explain the decreased accumulation of leukocytes and microglial cells observed at the injury site of CCI animals treated with trovafloxacin. A reduction in the number of pro-inflammatory cells at the ipsilateral side in CCI mice treated with trovafloxacin also correlates with the lower mRNA levels detected for pro-inflammatory cytokines (IL-1β, IL-6, and TNF-α) when compared to vehicle-treated CCI mice. Accumulation of activated pro-inflammatory microglia and macrophage cells can promote the release of various pro-inflammatory factors, which in turn are detrimental to neuronal health and eventually causes cell death [69, 70]. Microglial cells in the activated state, in the cortical area, persist for at least 1 year in animal models of TBI, indicating a chronic inflammatory process induced by brain trauma [71]. Indeed, the inflammatory-based progression of TBI in human postmortem studies shows that microglial activation remains for up to 17 years after TBI in subcortical brain areas [72, 73]. Thus, further studies are necessary to evaluate the role of Panx1 in the acute and chronic activation states of microglia and macrophage cells.

SBPD 120 kDa and MMP9, two well-known biomarkers associated with the worsening of brain injury after trauma, were found at high levels in vehicle-treated CCI mice, but were significantly reduced in trovafloxacin-treated CCI mice. SBPD 120 kDa is a byproduct of the neuronally expressed α–ΙΙ spectrin and is generated from sequential cleavage by caspase-3 proteases, which are activated upon neuronal injury and indicative of apoptotic cell death [74, 75]. Furthermore, pathological activation of MMPs, in particular MMP-9, has been shown to promote detrimental outcomes after brain injury, including blood brain barrier disruption, hemorrhage, and neuronal apoptosis [76, 77]. Hence, a reduction of the levels of MMP-9 in CCI mice treated with trovafloxacin might also contribute to the smaller hematomas observed in fixed brain from this group when compared to those that were treated only with vehicle.

To further link the actions of trovafloxacin with the blockade of Panx1 channel, we used Brilliant blue FCF another well-known Panx1 channel blocker [37]. As expected, Brilliant Blue FCF markedly reduced C5a-induced ATP release and migration in BV-2 cells in vitro further supporting a role for Panx1 channels. Importantly, Brilliant Blue FCF is a derivative of Brilliant Blue G; the latter has been shown to have anti-inflammatory and neuroprotective actions in mice and rats after traumatic brain injury [78, 79]. While Brilliant Blue G blocks both P2X7 channels and Panx1 channels, Brilliant Blue FCF only inhibits Panx1 channels [37]. Here, we found that mice treated with Brilliant blue FCF 1 h post-injury showed significant reduction of mRNA levels of pro-inflammatory cytokines IL-1β, IL-6, and TNF-α (Additional file 2: Figure S2). Lastly, our preliminary data show that mice treated daily with Brilliant Blue FCF (i.p. injection, 60 mg/kg) display a trend towards improved locomotor outcomes after 5 days post-injury (unpublished results). Unlike trovafloxacin, previous studies have shown that Brilliant Blue FCF is poorly absorbed from the gastrointestinal tract, and following absorption, it goes through extensive and rapid biliary and urinary excretion [80, 81]. Hence, further studies are necessary to find optimal doses and frequency of administration of Brilliant Blue FCF to establish a neuroprotective role in our model of brain trauma.

## Conclusions

Trovafloxacin treatment reduces inflammation and brain damage in a model of moderate TBI. We propose that the anti-inflammatory and neuroprotective actions of trovafloxacin are linked to the blockade of Panx1 channels due to the known roles of Panx1 channels in neuronal death during ischemia and infiltration of leukocytes during acute inflammation [9, 67, 11]. It is therefore possible that opening of Panx1 channels might potentiate the secondary damage response triggered by brain trauma. Future studies addressing the cell-type specific role of Panx1 in TBI will enhance our understanding and contribute to the development of Panx1 channels as a therapeutic target to improve outcome after brain injury.

## Additional files

Additional file 1: Figure S1. No detectable protein levels of MMP9, SPDBs, IgG, and CD68 in sham mice treated with trovafloxacin. Western blot analysis was performed to detect expression of SPDBs (140 and 120 kDa), MMP9, IgG, and CD68 in sham + trovafloxacin (TVX), sham + vehicle, and CCI-injured mice treated with vehicle. Only CCI-injured mice display protein expression of MMP9, SPDB120, IgG, and CD68 at the injury site. Bottom western blots below each protein marker correspond to GAPDH levels. Each lane corresponds to samples from different animals. (DOCX 771 kb)

Additional file 2: Figure S2. Blue Brilliant FCF treatment reduced CCI-induced pro-inflammatory cytokines. Administration of Blue Brilliant FCF via intraperitoneal injection (60 mg/kg) was performed 1 h post-CCI. Gene expression levels determined by qPCR for IL-1b, TNF-α, and IL-6 were measured 1 day post-injury. Values are expressed as mean fold change (± SEM) relative to sham (*n* = 8). GAPDH was used as an endogenous control. Statistical significance was evaluated using one-way ANOVA followed by Tukey’s HSD. (DOCX 85 kb)

## Abbreviations

ATP: Adenosine triphosphate
BBFCF: Brilliant Blue FCF
CCI: Controlled cortical impact
CD68: Cluster of differentiation
Cx: Connexin
DMEM: Dulbecco ‘s Modified Eagle Medium
GAFP: Glial fibrillary acidic protein
GAPDH: Glyceraldehyde 3-phosphate dehydrogenase
Iba1: Ionized calcium-binding adapter molecule 1
IL-1β: Interleukin-1β
IL-6: Interleukin-6
MMP9: Matrix metalloproteinase 9
MPO: Myeloperoxidase
Panx1: Pannexin 1
PBS: Phosphate-buffered saline
PFA: Paraforamaldehyde
TBI: Traumatic brain injury
TNF-α: Tumor necrosis factor alpha
TVX: Trovafloxacin
