# Supplementary material for: Trovafloxacin attenuates neuroinflammation and improves outcome after traumatic brain injury in mice
Source: J Neuroinflammation. 2018 Feb 13;15:42. doi: 10.1186/s12974-018-1069-9 (PMC5812039; doi:10.1186/s12974-018-1069-9)
Supplement: Supplementary file 1 — No detectable protein levels of MMP9, SPDBs, IgG, and CD68 in sham mice treated with trovafloxacin. Western blot analysis was performed to detect expression of SPDBs (140 and 120 kDa), MMP9, IgG, and CD68 in sham + trovafloxacin (TVX), sham + vehicle, and CCI-injured mice treated with vehicle. Only CCI-injured mice display protein expression of MMP9, SPDB120, IgG, and CD68 at the injury site. Bottom western blots below each protein marker correspond to GAPDH levels. Each lane corresponds to samples from different animals. (DOCX 771 kb) [file 12974_2018_1069_MOESM1_ESM.docx]

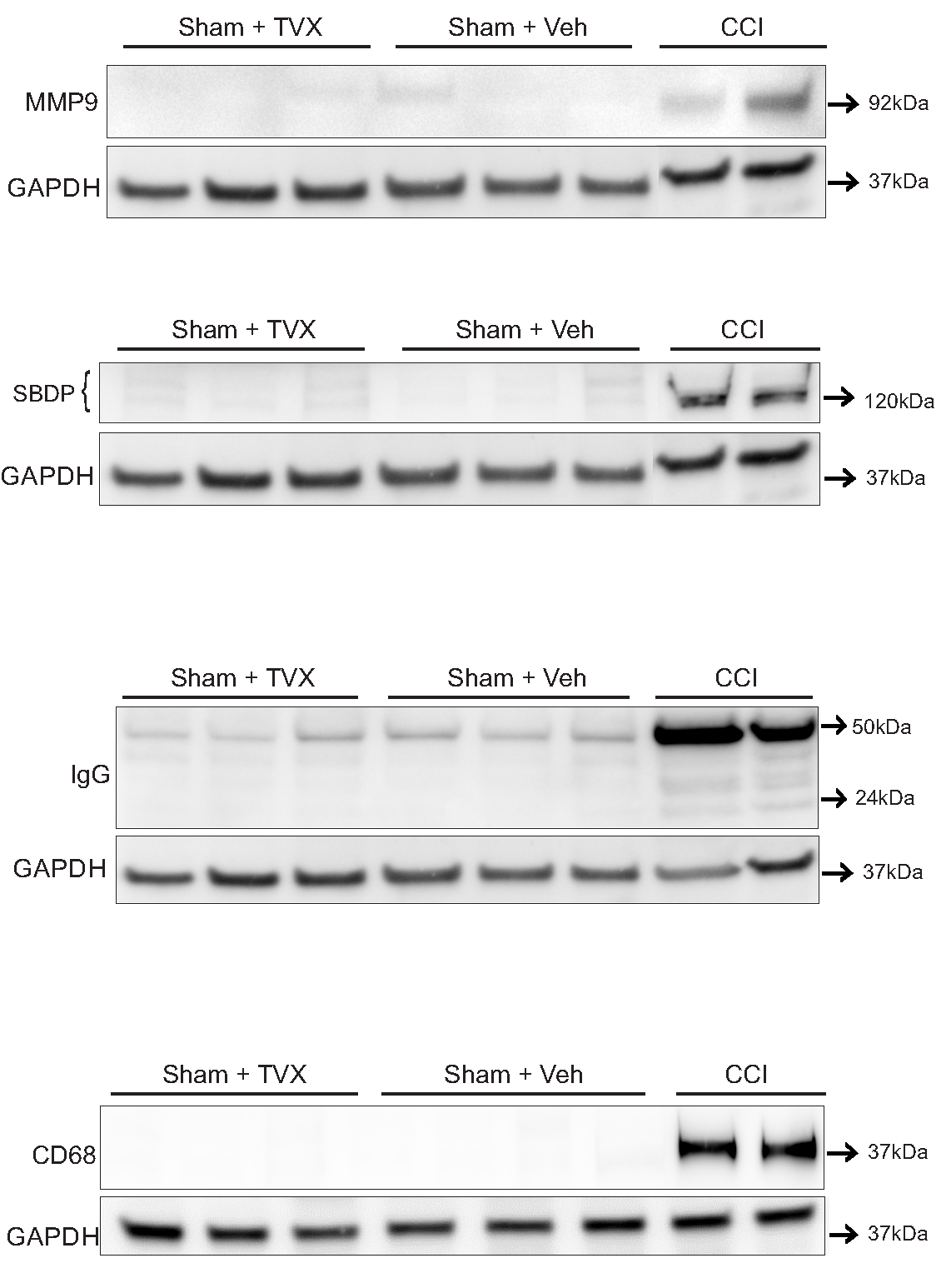


**Additional file 1: Figure S1. No detectable protein levels of MMP9, SPDBs, IgG and CD68 in sham mice treated with trovafloxacin.** Western blot analysis were performed to detect expression of SPDBs (140 and 120 kDa), MMP9, IgG and CD68 in sham + trovafloxacin (TVX), Sham + vehicle and CCI injured mice treated with vehicle. Only CCI injured mice display protein expression of MMP9, SPDB120, IgG and CD68 at the injury site. Bottom western blots below each protein marker correspond to GAPDH levels. Each lane corresponds to samples from different animals.
