# Supplementary material for: Trovafloxacin attenuates neuroinflammation and improves outcome after traumatic brain injury in mice
Source: J Neuroinflammation. 2018 Feb 13;15:42. doi: 10.1186/s12974-018-1069-9 (PMC5812039; doi:10.1186/s12974-018-1069-9)
Supplement: Supplementary file 2 — Blue Brilliant FCF treatment reduced CCI-induced pro-inflammatory cytokines. Administration of Blue Brilliant FCF via intraperitoneal injection (60 mg/kg) was performed 1 h post-CCI. Gene expression levels determined by qPCR for IL-1b, TNF-α, and IL-6 were measured 1 day post-injury. Values are expressed as mean fold change (± SEM) relative to sham (n = 8). GAPDH was used as an endogenous control. Statistical significance was evaluated using one-way ANOVA followed by Tukey’s HSD. (DOCX 85 kb) [file 12974_2018_1069_MOESM2_ESM.docx]

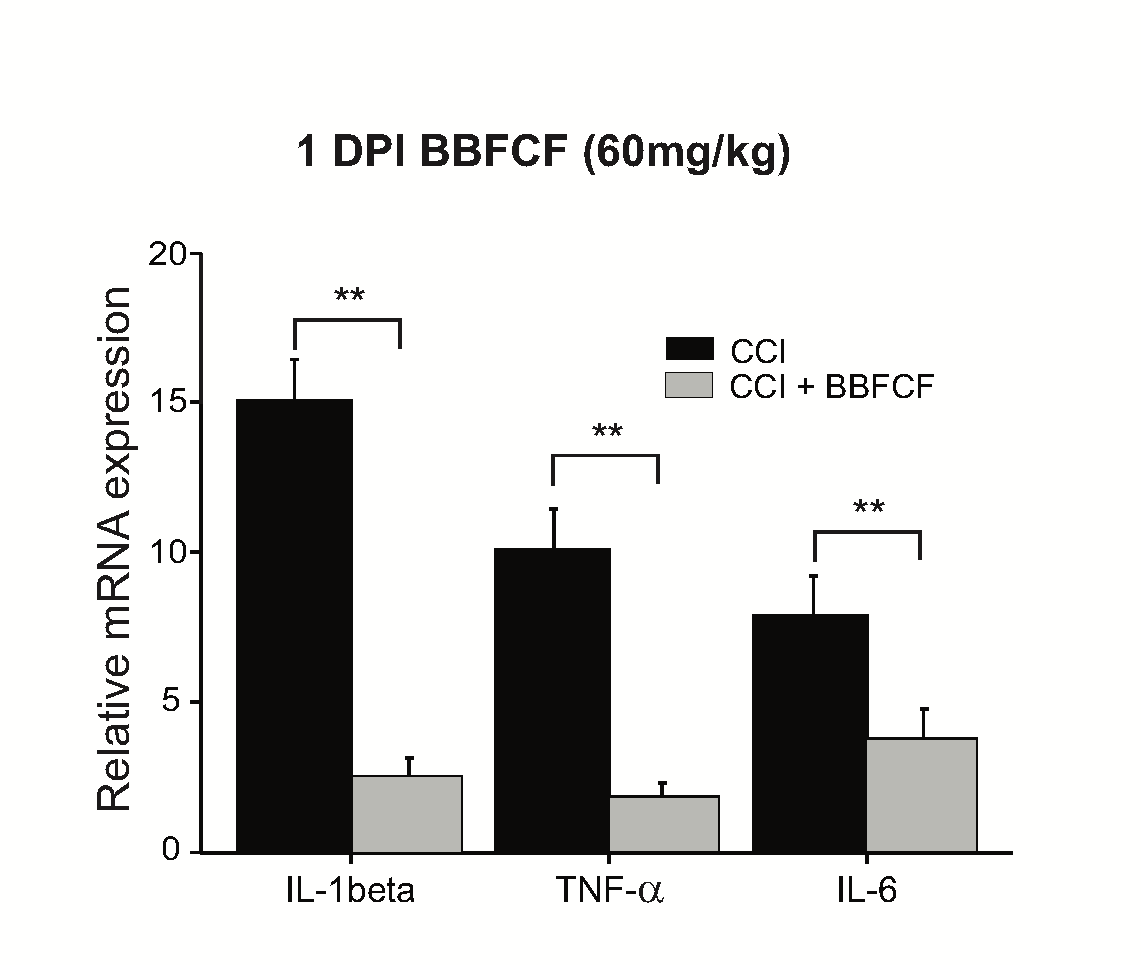


**Additional file 2: Figure S2.** Blue Brilliant FCF treatment reduced CCI-induced pro-inflammatory cytokines. Administration of Blue Brilliant FCF via intraperitoneal injection (60mg/kg) was performed one-hour post CCI. Gene expression levels determined by qPCR for IL-1β, TNF-α and IL-6 were measured 1 day post injury. Values are expressed as mean fold change (± SEM) relative to sham (n = 8). GAPDH was used as an endogenous control. Statistical significance was evaluated using One-way ANOVA followed by Tukey’s HSD.
